# Life on holidays: study protocol for a 3-year longitudinal study tracking changes in children’s fitness and fatness during the in-school versus summer holiday period

**DOI:** 10.1186/s12889-019-7671-7

**Published:** 2019-10-23

**Authors:** Amanda Watson, Carol Maher, Grant R. Tomkinson, Rebecca Golley, François Fraysse, Dorothea Dumuid, Hayley Lewthwaite, Tim Olds

**Affiliations:** 1 Alliance for Research in Exercise, Nutrition and Activity (ARENA), School of Health Sciences, University of South Australia, Adelaide, Australia; 2 Department of Education, Health and Behavior Studies, University of North Dakota, Grand Forks, USA; 3 College of Nursing and Health Sciences, Flinders University, Bedford Park SA 5042, South Australia

**Keywords:** Physical activity, Sedentary behaviour, Sleep, Diet, Use of time, Compositional data analysis, Obesity, Overweight, Cohort

## Abstract

**Background:**

Emerging evidence suggests that children become fatter and less fit over the summer holidays but get leaner and fitter during the in-school period. This could be due to differences in diet and time use between these distinct periods. Few studies have tracked diet and time use across the summer holidays. This study will measure rates of change in fatness and fitness of children, initially in Grade 4 (age 9 years) across three successive years and relate these changes to changes in diet and time use between in-school and summer holiday periods.

**Methods:**

Grade 4 Children attending Australian Government, Catholic and Independent schools in the Adelaide metropolitan area will be invited to participate, with the aim of recruiting 300 students in total. Diet will be reported by parents using the Automated Self-Administered 24-h Dietary Assessment Tool. Time use will be measured using 24-h wrist-worn accelerometry (GENEActiv) and self-reported by children using the Multimedia Activity Recall for Children and Adults (e.g. chores, reading, sport). Measurement of diet and time use will occur at the beginning (Term 1) and end (Term 4) of each school year and during the summer holiday period. Fitness (20-m shuttle run and standing broad jump) and fatness (body mass index *z*-score, waist circumference, %body fat) will be measured at the beginning and end of each school year. Differences in rates of change in fitness and fatness during in-school and summer holiday periods will be calculated using model parameter estimate contrasts from linear mixed effects model. Model parameter estimate contrasts will be used to calculate differences in rates of change in outcomes by socioeconomic position (SEP), sex and weight status. Differences in rates of change of outcomes will be regressed against differences between in-school and summer holiday period diet and time use, using compositional data analysis. Analyses will adjust for age, sex, SEP, parenting style, weight status, and pubertal status, where appropriate.

**Discussion:**

Findings from this project may inform new, potent avenues for intervention efforts aimed at addressing childhood fitness and fatness. Interventions focused on the home environment, or alternatively extension of the school environment may be warranted.

**Trial registration:**

Australia New Zealand Clinical Trials Registry, identifier ACTRN12618002008202. Retrospectively registered on 14 December 2018.

## Background

One in six children in OECD countries is classified as overweight or obese [1]. Overweight or obese children are at increased risk of developing non-communicable diseases [2], thereby establishing childhood overweight and obesity as a serious public health concern. There is also evidence showing that children’s aerobic fitness is worse today than several decades ago [3]. Childhood fatness as well as lower muscular and aerobic fitness track moderately well into adulthood, being associated with poorer adult health and higher mortality [4, 5]. Thus, strategies to address overweight and obesity and increase aerobic and muscular fitness levels during childhood are warranted.

Emerging research, predominantly conducted in North America, shows that increases in fatness and declines in aerobic fitness occur at a much greater rate during the school summer holiday period, compared with the school year [6–10]. These negative health outcomes are more pronounced among children who are already overweight or obese or from low socio-economic backgrounds [6]. Thus, the school summer holiday period is identified as a high-risk period for unfavourable changes in body composition, and declines in aerobic fitness, and may be an overlooked critical window for intervention.

Obesogenic behaviours, particularly children’s dietary intake (i.e. energy and nutrient intake, dietary pattern/quality [11]) and daily time use (i.e. time spent in physical activity, sedentary behaviour/screen time, sleep), as well as their environments (e.g. school and home), all play a role in facilitating or limiting increases in fatness or declines in fitness. Previous obesity interventions have predominantly been delivered in schools, and have had limited success [12]. The “Structured Days Hypothesis” provides a possible explanation for this, suggesting that obesity interventions may have more success if they target unstructured days (that is, days with few scheduled activities, such as school summer holidays), rather than the structured days (e.g. in-school period) [13]. The school day provides children with consistent structure, routine, and adult supervision, and provides all children with opportunities for favourable obesogenic behaviours. These include scheduled physical activity opportunities (e.g. physical education, recess and lunch), regulated caloric intake (e.g. school food and beverage availability, guidelines and/or legislation) and set meal/snack times, potentially limiting opportunities for unhealthy weight gain and declines in fitness [13]. In contrast, children may engage in more unfavourable obesogenic behaviours (e.g. poorer diet, more sedentary time and less physical activity) on less structured days (e.g. when at home during the school summer holidays). This could explain why children return to school fatter and less fit after the summer holidays [13].

There are few studies comparing children’s diet and time use during the school and summer holiday periods, and findings are mixed [14–18]. Some studies reported children had less favourable or similar diets during the school holidays, compared with the school year [14, 15], and engaged in more screen time during the school holidays (3.6 ± 1.5 h/day), compared with during the school term (3.1 ± 1.6 h/day) [15]. One study found that total daily energy expenditure (TDEE) during holidays (adjusted TDEE = 2450 ± 270 kcal/day) was not significantly different from TDEE during school term (adjusted TDEE = 2510 ± 350 kcal/day) among 6–13 year-olds at risk of overweight/obesity [16]. In contrast, others reported physical activity was higher during the school holidays, compared with school term [14, 15]. These studies are limited by the use of independent samples for school and holiday periods, and/or not specifically measuring the holiday period (i.e. weekend days were used as a proxy for school holidays), and by the omission of sleep duration [14–16]. In addition, they considered children’s activities such as screen time and physical activity as independent variables rather than as co-dependent parts of a 24-h day. If the time spent in some activities changes when children go on summer holidays, then the time spent in other activities must also change to compensate because there are only 24 h in a day. For a comprehensive exploration of changes in time-use behaviours, 24-h data are needed, and analytical methods appropriate for co-dependent variables should be used [19, 20].

One study of 366 Australian children compared 24-h time use on school days and holidays in the same children using analytical methods for co-dependent variables (compositional data analysis) [21]. During holidays, children slept an extra 40 min, accumulated 58 min more screen time and spent 10 min less in vigorous-intensity physical activity. Estimated TDEE was 5% lower. Further research is warranted to explore whether unfavourable changes in diet and time use occur over the summer holiday period.

### Aims and hypotheses

The focus of this research is to determine rates of change in fitness and fatness during in-school and summer holidays across successive school years, and to relate rates of change in these outcomes to changes in diet and time use. Specific hypotheses are:

1. Rates of change in fitness and fatness will differ between in-school and summer holiday periods.
2. The difference between in-school and summer holiday rates of change in fitness and fatness will differ according to the child’s socio-economic position (SEP), sex and weight status.
3. Differences in rates of change in fitness and fatness between in-school and summer holiday periods will be associated with differences in diet and time use between in-school and holiday periods.

## Methods/design

### Study design

A 3-year longitudinal study with rolling recruitment occurring over two years; participants will be followed for three successive years. Ethical approval has been obtained from University of South Australia Human Research Ethics Committee, Adelaide, Australia (200980), the South Australian Department of Education and Child Development (2008–0055) and the Adelaide Catholic Education Centre (201820).

### Participants and recruitment

Participants will be children, in Grade 4 (age approx. 9 years) at the time of enrolment, who attend a Government, Catholic or Independent primary school in the Adelaide metropolitan area. All Government, Catholic and Independent primary schools in the Adelaide metropolitan area (*n* = 334) will be manually categorised into tertiles according to their Index of Community Socio-Educational Advantage (ICSEA) score. The ICSEA provides an indication of socio-educational backgrounds of students and is based on parents’ occupation, parents’ education, geographical location and the proportion of indigenous students [22]. Schools will be randomly selected from tertiles with a probability proportional to the number of children enrolled. Selection of schools will continue progressively until at least 100 students in schools from each of low, middle and high tertiles have been recruited.

Principals from eligible schools will be invited to participate initially via email, and then be contacted via telephone one week later. A researcher will meet with all interested principals or their delegates to explain the requirements of study participation. Participating principals will be provided with a plain language statement and consent form to be signed and returned prior to commencement. All children in Grade 4 from each school will be invited to participate. Once consent is obtained from school principals, children in Grade 4 will be provided with an information pack containing an information sheet and consent form to take home to their parents/guardians. Written consent from parents prior to study participation, and verbal assent from children at each measurement occasion will be required. Assuming 24 students per class [23], 42 Grade 4 students per school [24] and a 50% response rate, approximately 14 schools will need to be recruited. As compensation for their time and commitment to the research project, at the end of each study year schools will receive $500 and the parents of each participating child will receive a $50 voucher. Each participating child will receive an age-appropriate gift at the conclusion of the study, as well as a small gift after each accelerometer administration to encourage return of the equipment.

### Measures

**Fig. 1:**
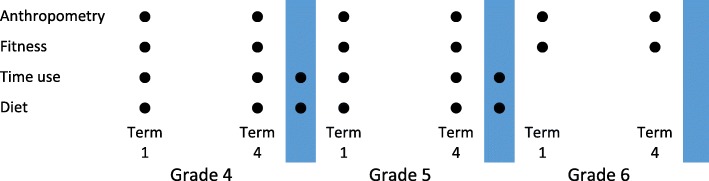
Schedule of assessments across the study period. The blue shaded areas indicate the holiday periods

The schedule of assessments across the study period is shown in Fig. 1. Diet and time use (accelerometry and 24-h recall) assessments will be conducted at the beginning (Term 1) and end (Term 4) of each school year and during each summer holiday period (December/January) for study years 1 and 2. Anthropometric and fitness measures will be taken at the beginning and end of each school year. Trained research personnel will administer fitness and fatness assessments in school, during usual school hours.

### Time use

Wrist-worn GENEActiv accelerometers will be used to provide a measure of children’s time spent sleeping, sitting and in light-, moderate- and vigorous- intensity physical activity. Accelerometers will be worn 24 h/day for 7 consecutive days. The GENEActiv has excellent convergent validity (r = 0.98) when compared to other accelerometers [25]. Intra-instrument and inter-instrument reliabilities are also strong (CV_intra_ = 1.4% and CV_inter_ = 2.1%), and it shows very good test-retest reliability (ICC = 0.67–0.87) [26]. The accelerometers will be distributed and collected from the children’s school at the beginning and end of each 7-day wear period and mailed to participants during school holiday periods with reply paid envelopes. A researcher will instruct children about accelerometer wear and care and provide children with an information leaflet to take home to their parents, following standardised protocols. Participants will also be provided with a paper self-report form where they will record their sleep and wake times, periods of device removal, along with the reason for removal. Data will be collected at 50 Hz and collapsed into 60-s epochs. The cut-points identified by Phillips et al. [27] will be used to identify durations of sedentary time, light- and moderate-vigorous physical activity. The algorithm proposed by van Hees et al. [28] will be used to derive sleep characteristics. Participants’ accelerometry data will be included in analyses if they wear the accelerometer for at least 10 waking hours, on at least three weekdays and one weekend day.

Specific types of activity will be captured using the Multimedia Activity Recall for Children and Adults (MARCA), a computerised 24-h recall [29]. Participating children will recall every activity (e.g. sports, reading, household chores) they did over a 2-day period during 30-min face-to-face interviews (terms 1 and 4) and during computer-assisted telephone interview (summer holidays). This will be done using a segmented-day format with a resolution of 5 min or more. Participants will choose from 500 activities, yielding a high-resolution snapshot of how they used their time. The MARCA has evidence of good validity (r = 0.4–0.7) when compared against accelerometry [29], pedometry [30] and doubly-labelled water [31], and excellent test-rest reliability (ICC = 0.88–1.00) [29]. Each activity is linked to a compendium of energy expenditures [32] so that overall and activity-specific energy costs can be estimated. Where possible, the same two days will be recalled at each time-point, including at least one weekday and one weekend day, overlapping where possible with GENEActiv data.

### Diet

Dietary intake will be assessed using the Automated Self-Administered 24 h Dietary Assessment Tool (ASA24), Australian version (2016), developed by the National Cancer Institute, Bethesda, MD, United States [33]. The ASA24 is an online tool for collecting 24-h dietary recall data using the seven-pass method (i.e. meal-based quick list, meal gap review, detail pass, forgotten foods, final review, last chance, usual intake) with digital photographic measures to aid portion size estimation [34, 35]. For children younger than 10 years, parent- or proxy-reported dietary intake is required [36, 37]. The ASA24 will be interviewer administered. Parent/carer proxy recalls will be undertaken via phone with the child present where possible so children can provide information on food and drinks consumed in the absence of the parent/carer. At each study time point, parents/carers will complete the ASA24 on one occasion (~ 30 min/recall), recalling food and drinks consumed over the previous 24-h. At the group level weekday and weekend days will be recalled. A second recall will be collected in a sub-sample of 10% to enable estimate of usual intake. Compared to dietary intake assessed via the plate wastage method, the ASA24 estimates energy intake within 0.52 kcal (95% CI − 236, 237) when self-administered by adults (i.e. parents/carer) [38]. Food group intake, energy and nutrient intake will be estimated using the Australian Food Supplement and Nutrient Database (AUSNUT) 2011–3 [39]. Usual energy and macronutrient intake will be derived using the web-based statistical modelling technique Multiple Source Method [40, 41].

Primary outcomes will be fitness and percentage body fat. Waist circumference and body mass index will be assessed as secondary outcomes.

### Fitness

Aerobic fitness will be assessed in schools using the 20-m shuttle run test [42]. This progressive exercise test involves continuous running between two lines 20 m apart. Audio signals pace the children, beginning at a speed of 8.5 km/h and increasing by 0.5 km/h every minute thereafter. The test ends when the child can no longer keep up with the pace of the beep for two consecutive laps. The shuttle run test provides a valid (r = 0.78) [43] and reliable (ICC = 0.78–0.93) [44] measure of aerobic fitness. Muscular fitness (explosive lower body strength) will be measured using a standing broad jump test. During this test, the child jumps as far forward as possible from a standing position [45], with the best of three jump attempts used in analyses. The standing broad jump test has very high test-retest reliability among children aged 5 to 12 years (ICC = 0.88) [46]. This test is also strongly associated with other lower body muscular strength tests (R^2^ = 0.83–86), as well as with upper body muscular strength tests (R^2^ = 0.69–0.85) [47]. Fitness will be measured using a composite score calculated as the average *z*-scores for the 20-m shuttle run and standing broad jump [48].

### Fatness

Percentage body fat will be measured via InBody 270 Bioelectrical Impedance Analyser (BIA) scales (InBody Co., Ltd., USA). The InBody BIA is a valid (r = 0.69–0.79 for children of this age) and reliable (CV_intra_ = 3%) estimate of body fat compared to underwater weighing [49].

Body mass index (BMI) will be derived from measured height and weight using the World Health Organisation Child Growth Standards [50]. Height and weight will be obtained using a Seca 213 stadiometer (Hamburg, Germany) and InBody BIA scales (InBody Co., Ltd., USA), respectively.

Waist circumference will be measured using a steel Lufkin W606 PM anthropometric tape held at the midpoint between the bottom of the rib cage and the top of the iliac crest. Both waist circumference and BMI have excellent intra- and inter-rater reliability (> 0.88 and > 0.90 respectively) [51]. All measures (percentage body fat, height, weight and waist circumference) will be taken twice. A third measure will be taken if there is more than 0.5 cm, 0.5 kg or 1.0% difference between the first and second measurements, with the mean of two or median of three measurements used in the analyses [52].

### Covariates

Covariates will include age, sex, SEP, parenting style, weight status (where appropriate) and pubertal status. Age, sex, SEP and parenting style will be obtained via a one-off parent questionnaire. Pubertal status will be obtained once per year via parent report.

- A composite SEP score will be derived from parent-reported occupation, household income and highest parental education level [53].
- Parenting style will be reported on three parenting dimensions (warmth, control and irritability) using questions derived from the Child Rearing Questionnaire [54] and the National Longitudinal Survey of Children and Youth [55], based on that used in a study by Wake and colleagues [56].
- Weight status will be obtained by categorising child BMI as either underweight, healthy weight, overweight or obese using the World Health Organisation Child Growth Standards [50].
- The Pubertal Development Scale will be used to provide a valid and reliable estimate of pubertal status [57]. For this, parents will be asked to report on their child’s stage of pubertal development based on a number of typical physical indicators associated with pubertal maturation, including the development of body hair, occurrence of growth spurt, and changes in complexion [58]. All questions will be answered on a 4-point scale (1 = has not begun, 2 = has barely started, 3 = is definitely underway, 4 = growth or development is definitely complete) [58].

### Power calculation

Required sample size has been calculated based on Hypothesis 1 (Rates of change in fitness and fatness will differ between in-school and summer holiday periods) using the gold standard of simulation for complex hierarchical models and verified empirically. Power calculations were checked against analytical power calculations for repeated measurements [59] and multi period cross-over designs [60]. Sample size calculations were performed using estimates of the variance components (the variance of random intercepts for schools, students and repeated measurements on students) for the outcome of percentage body fat (%BF), with the aim of being able to detect a meaningful difference in change of %BF of 0.6% per year between the in-school and holiday periods. A required sample size of 300 was estimated using Bonferroni corrected alpha of 0.025 for the two outcomes (fitness and BMI) in conjunction with a power of 0.8 and an assumed drop-out of 25% throughout the follow up.

### Analysis

Hypothesis 1 (Rates of change in fitness and fatness will differ between in-school [S] and summer holiday [H] periods) will be analysed using linear mixed effects modelling, with the rate of change in fitness and fatness across the in-school period (∆S) and holiday period (∆H) fitted as marginal fixed-slope effects, with age, sex and weight status (where appropriate), as covariates and random intercept and slope coefficients for student, class and school to account for repeated measurements and clustering at different levels of the hierarchy.

Differences between rates of change during in-school and holiday periods (∆_S–H_) will be calculated using model parameter estimate contrasts. We will test Hypothesis 2 (∆_S–H_ differs by SEP, sex and weight status) using similar model parameter estimate contrasts from the linear mixed effects models, with S–H as the dependent variable, and SEP, sex and weight status as the grouping factors (individually and conjointly), and age, sex and weight status where appropriate as covariates.

**Fig. 2:**
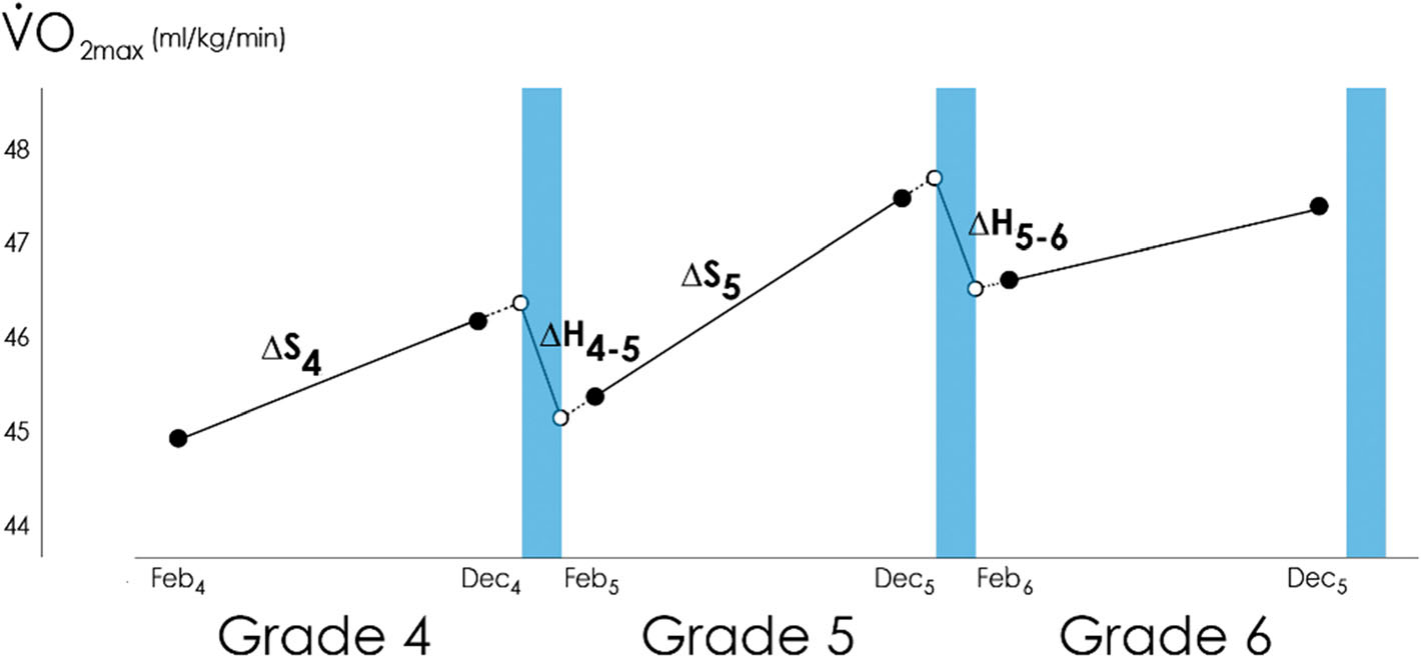
Mock data for changes in aerobic fitness. The blue shaded areas indicate the holiday periods. The black dots are measured values; the white dots are estimated fitness levels when extrapolated to the start, or back-extrapolated to the end of the holiday periods

Rates of change in outcomes during in-school periods (∆_S_) will be operationalised as the change per unit time between the February and December measurements. Rates of change in the summer holiday period (∆_H_) will be operationalised as the change per unit time between the point where the fitted line connecting the February and December measurements intersects with the start of the summer holidays, and the point where the fitted line connecting the February and December measurements of the following year intersects with the end of the summer holidays of the previous year (Fig. 2). This procedure estimates the unmeasured change between the December measurement and the end of the school term, and between the start of the school term and the February measurement.

Time use will be quantified as 24-h activity compositions [61]. These activity compositions will consist of mutually exclusive and exhaustive parts of the day which together sum up to 24 h. Accelerometry compositions will capture daily times spent in energy expenditure bands (sleep, sitting, light, moderate and vigorous PA), and MARCA compositions will capture times spent in activity “types” (e.g. chores, physical activity, school). The average daily accelerometry and MARCA time-use compositions will be used for analyses. Compositions will be expressed as isometric log ratios [62] to enable their inclusion in the linear mixed effects models. Differences in rates of change in outcome variables (S–H) will be regressed against differences between the in-school composition (C_S_) and the holiday composition (C_H_) (C_S–H_) to test Hypothesis 3 (∆_S–H_ is correlated with C_S–H_). Compositional isotemporal substitution analysis [63] will be used to quantify the change in fitness and fatness when replacing a given quantum of one component of the activity composition (e.g. 30 min of sitting) with the same quantum of another (e.g. 30 min of moderate- to vigorous- intensity physical activity), while holding the remaining components constant. A similar analysis will be performed for diet, with macronutrient mix or food group intake as the composition, and total energy intake as a covariate. Analyses will adjust for age, sex, SEP, parenting style, weight status, and pubertal status, where appropriate.

## Discussion

The focus of this study is to track changes in children’s fitness and fatness during the school year compared with the summer holidays across three successive school years, and to explore whether rates of change in these outcomes are associated with changes in diet and time use among Australian school children. There are policy implications if the holiday environment leads to increased fatness and decreased fitness. Potential interventions may target the home environment, and/or effectively extend the in-school environment. This could be achieved, for example, through use of family-based interventions, summer camps and summer school programs. Summer camps and programs offer a mix of physical and specialised learning activities and may provide the structured day needed to prevent weight gain and losses in fitness.

There is strong evidence that summer camps, with appropriate activity components, can be effective in promoting physical activity [64, 65]. While there is little research, it has also been suggested that summer camps may provide a valuable setting for interventions aimed at improving children’s diet over the summer holiday period as they provide a structured environment where camp administrators determine the food provided to children [66]. Increased summer holiday physical activity and improved diet may translate into lower fatness and higher fitness [67–69].

Family-based interventions may also provide a strategy to prevent unhealthy changes in health outcomes. Although challenging, especially during the holiday diaspora, family-based interventions can be effective in increasing physical activity, with a recent systematic review [70] showing a small-to-moderate benefit (ES = 0.29). A further systematic review found family-based interventions were more effective than school-based interventions for reducing obesity in children under the age of 12 [71].

## Conclusions

Emerging evidence suggests that the summer holiday period is characterised by increased fatness and decreased fitness. It is possible that these patterns may be due to changes in diet and/or time use (e.g. children may be less active, eat more and have more screen time during the school holidays, compared with the in-school period). However, few studies have tracked diet and time use across the holiday period. Thus, the focus of this project is to track changes in fitness, fatness, diet and time use of 9–11-year-old children across three successive years and compare rates of change between in-school and summer holiday periods. Findings from this project are likely to inform new, potent avenues for intervention efforts aimed at addressing childhood fitness and fatness.

## Data Availability

The datasets will not be publicly available due to ethical restrictions (participants have not consented to the use of their data for purposes other than those for which they originally consented). An ethically compliant dataset could be made available upon reasonable request to the corresponding author.

## Abbreviations

%BF: percentage body fat
ASA24: Automated Self-Administered 24 h Dietary Assessment Tool
AUSNUT: Australian Food Supplement and Nutrient Database
BIA: Bioelectrical Impedance Analyser
BMI: Body mass index
ICSEA: Index of Community Socio-Educational Advantage
MARCA: Multimedia Activity Recall for Children and Adults
SEP: socio-economic position
TDEE: total daily energy expenditure
